# Altered whole-brain gray matter volume in form-deprivation myopia rats based on voxel-based morphometry: A pilot study

**DOI:** 10.3389/fnins.2023.1113578

**Published:** 2023-04-18

**Authors:** Jiayan Liu, Yahui Lei, Yuyao Diao, Yamei Lu, Xingbo Teng, Qingting Chen, Lian Liu, Jingxiang Zhong

**Affiliations:** ^1^Department of Ophthalmology, First Affiliated Hospital of Jinan University, Jinan University, Guangzhou, China; ^2^Department of Ophthalmology, The Sixth Affiliated Hospital of Guangzhou Medical University, Qingyuan People’s Hospital, Qingyuan, China; ^3^Medical Imaging Center, First Affiliated Hospital of Jinan University, Guangzhou, China; ^4^The Sixth Affiliated Hospital of Jinan University, Jinan University, Dongguan, China

**Keywords:** gray matter volume, voxel-based morphometry, FDM, rat, magnetic resonance imaging

## Abstract

**Background:**

Myopia is one of the major public health problems worldwide. However, the exact pathogenesis of myopia remains unclear. This study proposes using voxel-based morphometry (VBM) to investigate potential morphological alterations in gray matter volume (GMV) in form-deprivation myopia (FDM) rats.

**Methods:**

A total of 14 rats with FDM (FDM group) and 15 normal controls (NC group) underwent high-resolution magnetic resonance imaging (MRI). Original T2 brain images were analyzed using VBM method to identify group differences in GMV. Following MRI examination, all rats were perfused with formalin, and immunohistochemical analysis of NeuN and c-fos levels was performed on the visual cortex.

**Results:**

In the FDM group, compared to the NC group, significantly decreased GMVs were found in the left primary visual cortex, left secondary visual cortex, right subiculum, right cornu ammonis, right entorhinal cortex and bilateral molecular layer of the cerebellum. Additionally, significantly increased GMVs were found in the right dentate gyrus, parasubiculum, and olfactory bulb.

**Conclusions:**

Our study revealed a positive correlation between mGMV and the expression of c-fos and NeuN in the visual cortex, suggesting a molecular relationship between cortical activity and macroscopic measurement of visual cortex structural plasticity. These findings may help elucidate the potential neural pathogenesis of FDM and its relationship to changes in specific brain regions.

## 1. Introduction

Myopia has become one of the major public health problems worldwide (Flaxman et al., 2017). Approximately 50% of the population in Asia suffers from myopia. The number of people with myopia is expected to increase to 4.95 billion (52%) by 2050 (Holden et al., 2016). High myopia can lead to serious vision-threatening complications, including myopic choroidal neovascularization, retinal detachment, and an increased risk of glaucoma and cataract (Gao et al., 2011; Kang and Koh, 2014; Choquet et al., 2022). Myopia can seriously affect the quality of life of patients, and bring a heavy economic burden to their families and society (Ma et al., 2022). However, the exact pathogenesis of myopia is not yet clear, and the high incidence rate of myopia is mainly due to a variety of environmental and genetic factors (Jones et al., 2007).

In recent years, MRI technology has become a valuable tool for studying brain function. This includes various techniques such as structural MRI, functional MRI that detects blood oxygen saturation levels (BOLD-fMRI), arterial spin labeling imaging (ASL), Diffusion Tensor Imaging (DTI), and diffusion kurtosis imaging (DKI). These methods, which include tract-based spatial statistics analysis based on DTI data (TBSS), voxel-based morphometry (VBM) based on high-resolution structural imaging, tfMRI and rs-fMRI based on BOLD, have provided non-invasive, high-resolution insights into changes in brain morphology, microstructure, and functional activity in different regions of the brain. They have been widely used in both basic and clinical research. However, rs-fMRI analysis is susceptible to motion artifacts, which requires processing methods like motion correction and artifact removal to reduce errors and improve accuracy (Friston et al., 1996). Additionally, rs-fMRI data is prone to errors and false positives due to various factors, including physiological noise, motion, and cerebral blood flow. Its spatial resolution is relatively low, making it challenging to analyze small structures or details (Sporns, 2014). VBM, on the other hand, is a new technique for accurately studying brain morphology using voxel analysis of brain structure MRI. VBM allows for the whole brain to be measured and compared, enabling the direct analysis of raw data without prior assumptions of the region of interest. This method quantitatively detects changes in gray and white matter density and volume of local brain tissue and is not subject to researchers’ subjective influence (Boscolo Galazzo et al., 2020). It offers autonomy, comprehensiveness, objectivity, and repeatability advantages (Ashburner and Friston, 2001; Draganski et al., 2011). VBM facilitates the identification of brain areas associated with specific disorders, advancing our understanding of the structural changes caused by those diseases. Recently, the VBM approach has been successfully used in many ocular diseases, such as age-related macular degeneration (Ge et al., 2021), blindness (Pan et al., 2007), and primary angle-closure glaucoma (Jiang et al., 2018). The technique has been successfully demonstrated to be reliable and credible in clarifying pathophysiological pathways and identifying aberrant anatomy in ophthalmological illnesses. These investigations have demonstrated that alterations in gray matter (GM) or white matter (WM) volume or concentration are closely related to alterations in brain function and the onset of disease. Diverse diseases alter different brain regions, suggesting that region-specific brain changes can be used to determine a disease’s onset or course. Future pathological studies can benefit from pinpointing the exact location of these alterations.

Recent studies have increasingly utilized MRI to investigate the ocular morphology of myopia patients, suggesting that the condition may be linked to vision loss (Yu et al., 2018). Three-dimensional MRI studies provide valuable qualitative insights into myopia and posterior staphyloma based on objective and quantitative measurements (Guo et al., 2017). Visual deprivation can cause abnormal function of the visual cortex and vision-related cortex, and lead to plastic changes in the brain’s visual system (Liu et al., 2020). Using the VBM method, Takeuchi (Takeuchi et al., 2018) found that refractive errors appear to be mainly associated with the volume of the cranium. Additionally, Li et al. (2012) reported an increased concentration of WM in patients with high myopia, primarily in the calcarine area and the prefrontal and parietal lobes. Meanwhile, Liu discovered abnormal spontaneous alterations in WM of monocular blindness patients (Liu et al., 2020). However, there is currently limited research on brain morphology regarding myopia onset during the sensitive period of development.

FDM can be complicated by congenital cataract, ptosis, corneal leukoma and other eye diseases that can result in irreversible visual impairment if not treated properly. It is commonly believed that this irreversible injury is related to retrobulbar lesions; however, the specific neural mechanisms are not yet completely understood due to the difficulty in obtaining brain tissue samples for such diseases. Instead of using human subjects, animal imaging studies can use invasive methods such as histology, electrophysiology, or molecular biology to verify the results obtained from non-invasive methods such as MRI. Additionally, animal imaging studies can use these invasive methods to investigate the underlying mechanisms of the observed changes in brain structure or function, which is not feasible or ethical in human studies. FDM rats are widely used animal models of myopia that can simulate the main characteristics of human myopia, such as axial elongation, reduced refractive error, and altered ocular biometry (Tkatchenko et al., 2010). Studies have shown discovered that genes related to animal myopia overlap considerably with genes near the human myopia gene locus, demonstrating that the pathophysiology of myopia is comparable in animal and human models (Vutipongsatorn et al., 2019). Therefore, using FDM rats allows us to explore the neural correlates and causes of myopia more comprehensively and rigorously than using humans. Additionally, using FDM rats allows us to control for confounding factors such as age, sex, genetic background, and environmental influences that may affect the results in human studies (Chen et al., 2022; Wahyuningsih et al., 2022). In this study, we utilized a VBM approach to evaluate the changes in the structure of the rat brain caused by monocular form deprivation. We investigated the underlying neural mechanisms of myopia and searched for new opportunities for the prevention and treatment of myopia by studying the changes and differences in brain structure between the form-deprivation myopia (FDM) group and the normal control (NC) group.

## 2. Materials and methods

### 2.1. Animal groups and experimental design

A total of 35 Sprague Dawley rats, aged 3 weeks, were housed in a specific-pathogen-free environment under controlled conditions and provided with adequate food and water. The experiment was approved by the Laboratory Animal Ethics Committee of Jinan University, China. The rats were divided into two subgroups: the NC group (n = 15) and FDM group (n = 20). The FDM group was used to create form-deprivation myopia. Then, the right eyelids of the rats were sutured using 5–0 nylon to create form deprivation for 4 weeks. The eyelids were sutured under general anesthesia by intraperitoneal injection of 2% pentobarbital (40 mg/kg). The eyelids were checked daily to ensure that they were completely closed. The left eyes were left open and used as the controls. The refractive errors of both eyes were measured before and 4 weeks after monocular form deprivation. After 4 weeks, the rats were killed after anesthesia administration, and their eyeballs were removed to measure the axial length using a vernier caliper. Rats were excluded if the difference in their axial length between the right and left eye was less than 0.1 mm. A final total of 14 rats in the FDM group and 15 rats in the NC group were included in this study (Table 1).

**Table 1:** The baseline of characteristics of rats in the study.

Variable	FDM group	NC group	*t*/*x*^2^	*p*
Age(weeks)	3	3	NA	>0.999
Sex			0.024	0.876
Male	6 (42.86%)	6 (40.00%)		
Female	8 (57.14%)	9 (60.00%)		
Weight (g)	67.20 ± 2.57	67.80 ± 2.37	0.598	0.555
Diopter (R)	7.32 ± 0.49	7.33 ± 0.48	0.107	0.916
Diopter (L)	7.17 ± 0.36	7.20 ± 0.25	0.292	0.772

R, right eye; L, left eye; FDM, form-deprivation myopia; NC, normal control.

### 2.2. Measurements of refractive errors

Their pupils were dilated using three drops of 0.5% tropicamide/phenylephrine before measurements were performed. Two masked orthoptists used streak retinoscopy (Liuliu, Suzhou, China) and trial lenses to measure the refractive errors. The refractive errors were calculated by taking the average of the spherical components of the horizontal and vertical meridians.

### 2.3. MRI acquisitions

MRI examinations were performed using a preclinical BioSpec 9.4 T scanner (Bruker, Ettlingen, Germany) running on ParaVision 360 V1.1. The signal was generated using an 86-mm quadrature volume resonator and a 38-mm four-channel (2 × 2) single loop surface coil for reception. Initial induction of anesthesia was performed using 3% isoflurane, and 1–1.5% isoflurane (with 2 l/min of oxygen flow) was used to maintain it throughout the MRI procedure. The rats were placed in a prone position, head first in the scanner, and a tooth rod was used to fix their heads. A respiratory rhythm sensor was placed under the abdomen to track and observe respiration at a rate of 50–80 breaths per minute in real-time. To prevent the rats’ body temperature from dropping while undergoing MRI, they were kept on an animal heating pad connected to hot circulating water at 37°C ± 1.0°C. A T2-weighted RARE anatomical image was acquired with the following parameters: rare factor = 8, TR = 6,000 ms, TE = 22.28 ms, an acquisition matrix of 150 × 105 with 70 axial slices, slice thickness = 0.4 mm, in-plane resolution of 0.2× 0.2 mm^2^ and FoV = 30 × 21 mm^2^ covering the entire brain.

### 2.4. MRI data preprocessing

T2-weighted imaging was performed using the SPM8 toolbox (Statistical Parametric Mapping; Wellcome Centre for Human Neuroimaging, London, UK)^1^ and MATLAB (MathWorks Inc., Natick, MA, USA, version 8.4, 2014b). Preprocessing included the following steps: first, the anatomical voxel size was scaled up by a factor of 10 to account for the smaller brain volume of rodents and optimize their use in standard neuroimaging software packages designed for human imaging (Magalhães et al., 2017). Then, the scaled T2-weighted images were manually reoriented one by one to the origin of the anterior commissure. After that, the structural images were segmented into GM, WM, and cerebrospinal fluid areas based on the prior of SIGMA rat brain template (Sumiyoshi et al., 2014) using the unified standard segmentation option in SPM8. After segmentation, the individual GM components were normalized into the standard SIGMA rat brain template. Finally, the images were smoothed with a 5-mm full-width at half-maximum Gaussian kernel. Voxel-wise differences between the two groups were compared while adjusting for sex and relative total intracranial volume (Gaussian random field corrected). The significantly altered clusters in the GM were saved as masks, and their corresponding mean gray matter volume (mGMV) was extracted using the Dpabi toolbox.

### 2.5. Immunohistochemistry (IHC) for proteins in the visual cortex

At the fourth week following monocular form-deprivation, the visual cortex of rats in both groups was dissected according to “the rat brain in stereotaxic coordinates” and immediately fixed in a 4% paraformaldehyde solution. The tissue was then dehydrated, embedded, and sectioned to a thickness of 4 μm using a paraffin microtome. The sections were dewaxed, hydrated, and subjected to microwave antigen retrieval after treatment with 3% H_2_O_2_ to eliminate endogenous peroxidase activity. After blocking with a 5% BSA blocking solution, primary antibodies including Anti-c-Fos Mouse and Anti-NeuN Rabbit were applied, followed by a secondary antibody HRP-labeled goat anti-rat Ig. The sections were developed with DAB, counterstained with hematoxylin, dehydrated, mounted, and imaged using a microscopic imaging system. The integrated optical density (IOD) was analyzed using the IHC plug-in of ImageJ software. Pearson correlation analysis was performed between the IOD of NeuN and c-fos expression and mGMV.

### 2.6. Statistical analysis

After adjusting for relative total intracranial volume and sex, a general linear model analysis with the SPM8 toolbox was performed to compare the GM between the FDM group and NC group. Gaussian random field (GRF) theory was used for large-scale comparison rectification (GRF calibrated, voxel standard *p* < 0.005, cluster standard *p* < 0.05). Other data analyses were performed using GraphPad Prism (version 8.3; GraphPad, La Jolla, CA). The two-sample independent t-test was used to compare ocular diopter and axis length between the two groups. The chi-squared test was used to compare categorical variables between the two groups. The positive areas of IHC staining were analyzed by ImageJ. The IOD was used in our research, which accurately reflects the total protein expression in IHC staining (Du et al., 2016). Data were presented as mean ± standard deviation.

## 3. Results

### 3.1. Refractive results

**Figure 1:**
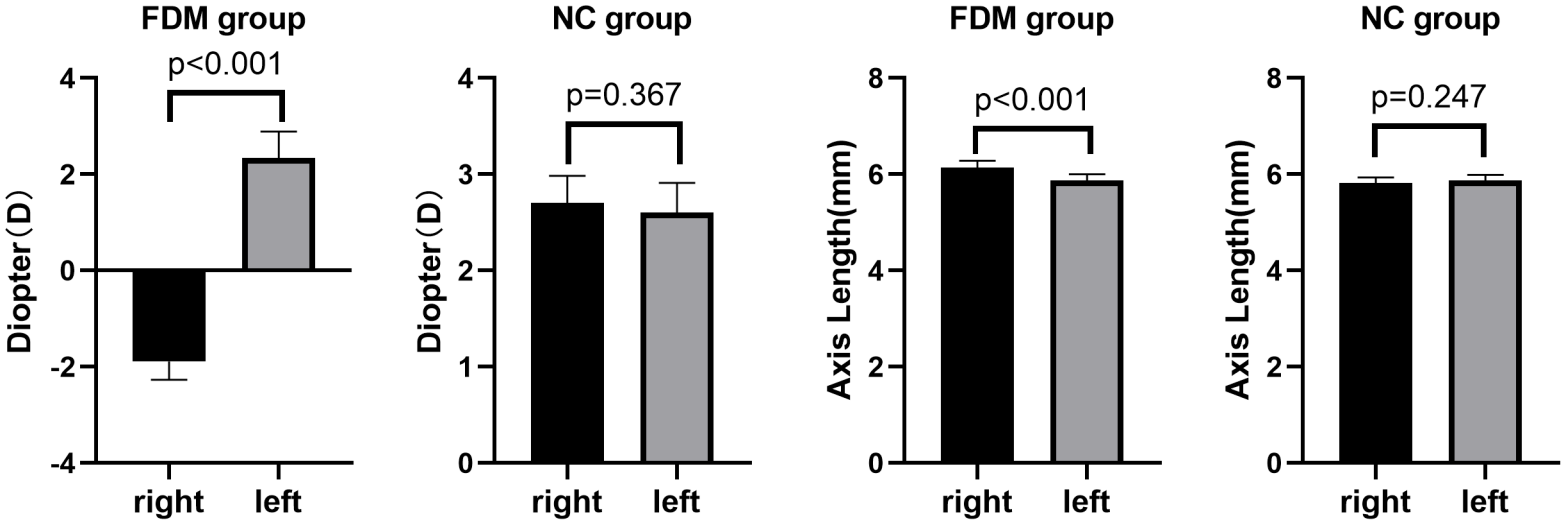
Comparison of binocular axial length and diopter in the two groups. In the FDM group, the mean diopter of the right eye was −1.89D and that of the left eye was 2.34D. In the NC group, the mean diopter of the right eye was 2.70D and that of the left eye was 2.60D. In the FDM group, the mean axis length of the right eye was 6.11 mm and that of the left eye was 5.84 mm. In the NC group, the mean axis length of the right eye was 5.82 mm and that of the left eye was 5.87 mm. FDM, form-deprivation myopia; NC, normal control.

The FDM group was modeled as previously described in the experimental design (Shinohara et al., 2018). Rats were excluded from the study if the difference in axial length between their right and left eyes was less than 0.1 mm or if the ocular diopter of the model eyes was > − 0.50D (Baird et al., 2020). A total of 14 rats in the FDM group and 15 rats in the NC group were included in this study (Table 1). Significant differences inocular diopter and axial length were observed between the form deprivation eye and the fellow eye in the FDM group. However, in the NC group, no significant differences in ocular diopter and axial length were found between the two eyes (Figure 1). In the FDM model eyes, the diopter was decreased and the axial length was increased compared to the eyes of the NC group, indicating that the FDM model was successfully established.

### 3.2. VBM difference

**Figure 2:**
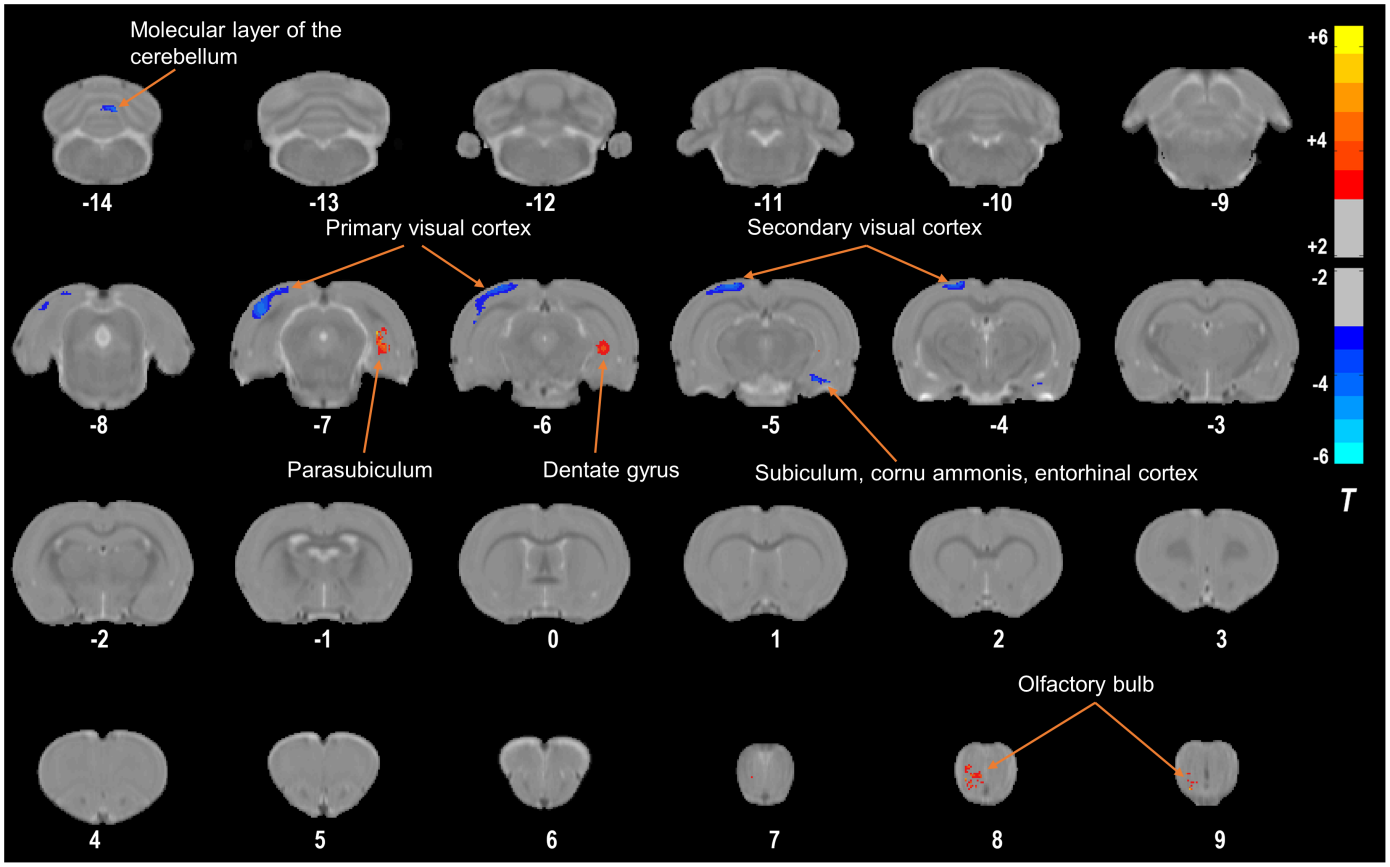
Compared with the NC group, the FDM group had altered GMVs. The regions with decreased GMVs are shown in blue, including the left primary visual cortex, left secondary visual cortex, right subiculum, right cornu ammonis, right entorhinal cortex, and bilateral molecular layer of the cerebellum. The regions with increased GMVs are shown in red, including the right dentate gyrus, parasubiculum, and olfactory bulb. NC, normal control; FDM, form-deprivation myopia; GMV, gray matter volume.

**Figure 3:**
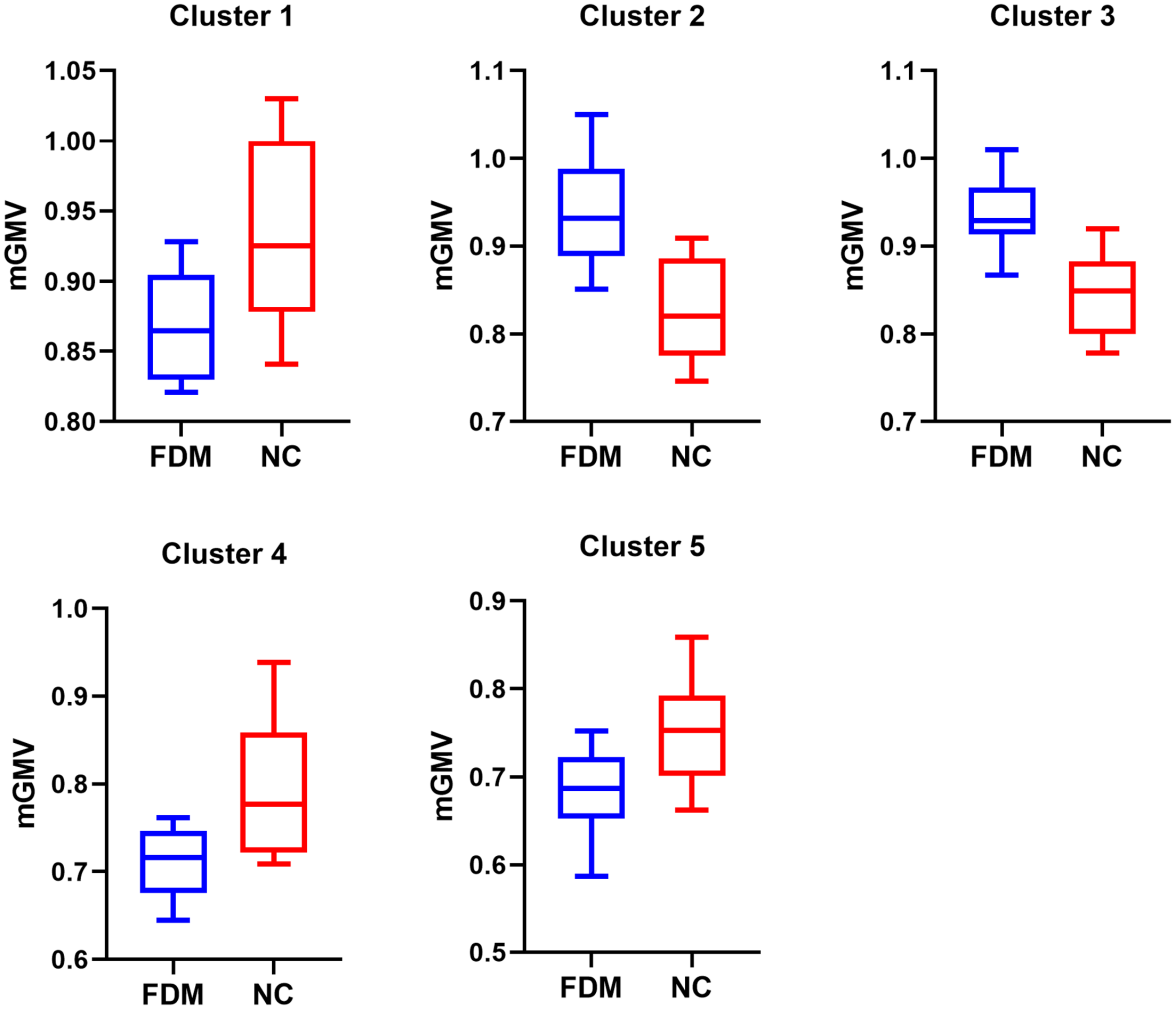
The significant GMVs between the FDM group and NC group. The left denotes the FDM group, and the right denotes the NC group in five significantly different clusters. GMV, gray matter volume; FDM, form-deprivation myopia; NC, normal control.

Using a VBM approach, we found that compared with those in the NC group, 4 weeks of right eye form deprivation led to a decrease in GMVs of the left primary visual cortex, left secondary visual cortex, right subiculum, right cornu ammonis, right entorhinal cortex, and bilateral molecular layer of the cerebellum (Figure 2; Table 2; for a full list of significant GRF correction, voxel-level *p* < 0.005, cluster-level *p* < 0.05). By contrast, FDM rats exhibited significantly increased GMVs in the right dentate gyrus, parasubiculum, and olfactory bulb (Figure 2; Table 2). And the main functions of the brain regions involved in this study were listed in Table 3. Moreover, we demonstrated the altered mGMV changes between the two groups using a box plot (Figure 3).

**Table 2:** Differences in GMV between the FDM and NC groups.

Index	Brain regions	Hemisphere	Cluster size (voxels)	Peak T values	Peak coordinates^a^		
					*x*	*y*	*z*
FDM < NC							
Cluster 1	Primary visual cortex, secondary visual cortex	L	2,753	−4.92	−3.45	−4.61	6.74
Cluster 4	Subiculum, cornu ammonis, entorhinal cortex	R	235	−4.74	3.45	−4.31	−13.65
Cluster 5	Molecular layer of the cerebellum	R/L	199	−5.08	0.90	−14.51	2.84
FDM > NC							
Cluster 2	Dentate gyrus, parasubiculum	R	681	5.77	4.50	−7.01	2.39
Cluster 3	Olfactory bulb	L	442	5.95	−1.95	7.84	2.09

GMV, gray matter volume; FDM, form-deprivation myopia; NC, normal control.

a The coordinates based on the SIGMA rat brain atlas and the origin point is Anterior Commissure.

**Table 3:** The main functions of the brain regions involved in this study.

Brain structure	Function	Reference
Primary visual cortex	Receives, integrates, and processes visual information from the retina	Huff et al. (2022), Tong et al. (2021)
Secondary visual cortex	Processes complex aspects of vision such as motion, color, shape, and depth	Huff et al. (2022), Shipp and Zeki (1989)
Subiculum	Involved in memory processing, learning, spatial navigation, and emotions	Chauhan et al. (2021), O’Mara et al. (2001)
Cornu ammonis	Involved in memory formation and consolidation	Chauhan et al. (2021), Shang et al. (2023)
Entorhinal cortex	Provides input to the hippocampus and receives output from it; involved in memory and navigation	Chauhan et al. (2021), Hisey et al. (2023)
Molecular layer of the cerebellum	Contains interneurons that modulate the activity of Purkinje cells; involved in motor coordination and learning	Kim and Augustine (2021)
Dentate gyrus	Involved in memory formation and pattern separation	Chauhan et al. (2021), Jeremic et al. (2023)
Parasubiculum	Involved in spatial orientation and navigation	Chauhan et al. (2021), Wan et al. (2020)
Olfactory bulb	Receives sensory input from the olfactory receptors; involved in odor detection and discrimination	Wang et al. (2023), Akar et al. (2023)

### 3.3. Protein expression of NeuN and c-fos in visual cortex by IHC

**Figure 4:**
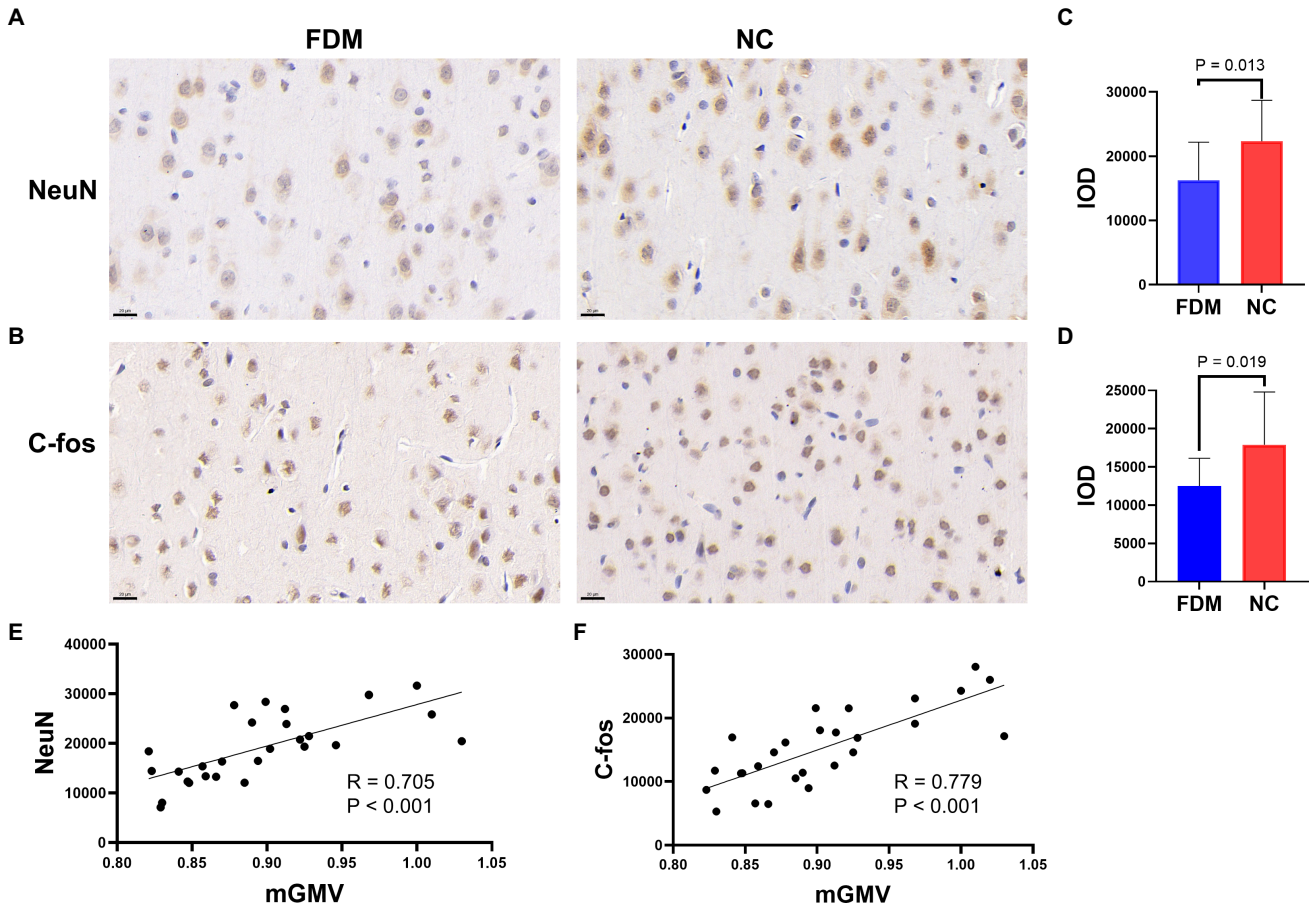
NeuN **(A)** and c-fos **(B)** expression in the tissues of visual cortex by immunohistochemistry in the FDM group and NC group (scale bar: 20 μm). Histograms showing the IOD of NeuN **(C)** and c-fos **(D)** expression in the FDM group and NC group (*p* < 0.05). Plot of the significant correlation between IOD of NeuN expression and mGMV of visual cortex **(E)**. Plot of the significant correlation between IOD of c-fos expression and mGMV of visual cortex **(F)**. NC, normal control; FDM, form-deprivation myopia; mGMV, mean gray matter volume; IOD, integrated optical density.

The IHC results showed positive signals of NeuN and c-fos in the visual cortex of rats in both groups after 4 weeks of form-deprivation. The IOD of NeuN and c-fos expression in the visual cortex of the FDM group was significantly lower than that of the NC group (Figure 4). The IOD of NeuN expression was positively correlated with the mGMV of the visual cortex in Cluster 1 (*R* = 0.705, *p* < 0.001), and the IOD of c-fos expression was positively correlated with the mGMV of the visual cortex in Cluster 1 (*R* = 0.779, *p* < 0.001) (Figure 4).

## 4. Discussion

The purpose of this study was to investigate neuroanatomical changes in a rat model of FDM using the technology of VBM. Compared with traditional morphometric analyses using anatomically defined regions of interest (Kuroki et al., 2006) and surface-based methods that only detect cortical thickness and sulcus depth (Tosun et al., 2015), the VBM approach is faster and provides more information about the brain.

**Figure 5:**
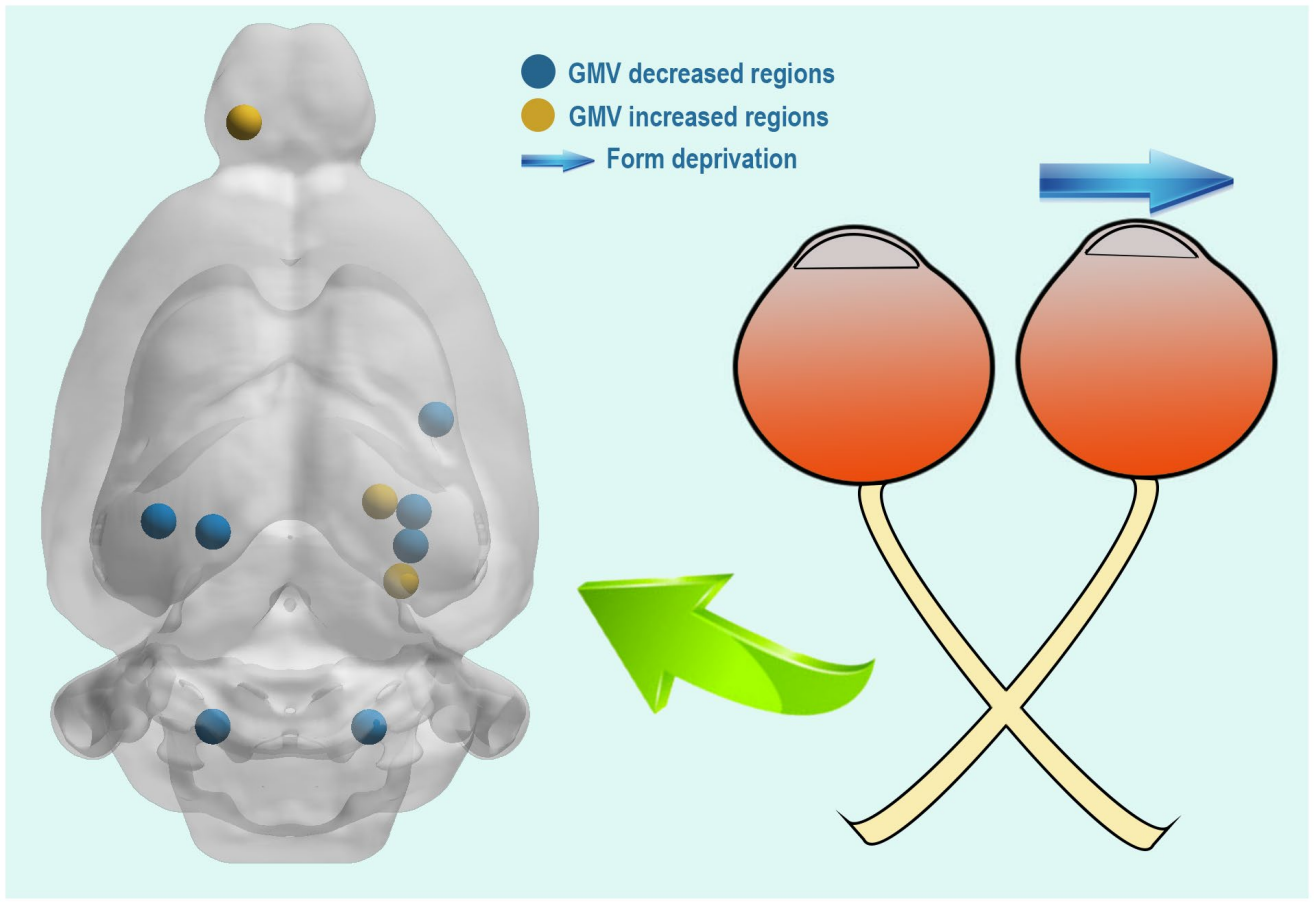
Compared with the NC group, the FDM group had altered brain regions after 4 weeks of right eye form deprivation. NC, normal control; FDM, form-deprivation myopia.

To our knowledge, this is the first study to investigate GMV changes in FDM rats using a VBM approach. VBM analysis involves a voxel-by-voxel comparison of the local GM concentration (Ridgway et al., 2008). In this study, we observed that compared to the NC group, FDM rats had a significant decrease in GMVs in the left primary visual cortex, left secondary visual cortex, right subiculum, right cornu ammonis, right entorhinal cortex, and bilateral molecular layer of the cerebellum. However, they had significantly increased GMVs in the cluster of the right dentate gyrus, parasubiculum, and olfactory bulb (Figure 5).

Our study found that GMVs decreased in the contralateral primary visual cortex and secondary visual cortex of the form-deprived eyes of rats. This finding is closely related to the anatomy of the optic chiasm in rats, as their visual system differ from that of ordinary mammals. In rats, most of the optic nerves pass through the optic chiasm to the visual cortex of the opposite brain. Unilateral visual deprivation in newborn rats results in changes and structural alterations in many parts of the visual system, with hemicerebral selectivity. That is, it affects the opposite hemibrain of the deprived eye. As early as the 1960s, Hubel and Wiesel (Wiesel and Hubel, 1963; Hubel and Wiesel, 1964) studied the response of the primary visual cortex of kittens to image stimuli following monocular form deprivation that severed the visual conduction pathway in kittens. Similarly, Takahata et al. (2018) discovered that abnormal binocular interaction accelerates visual cortex plasticity in monocular deprivation macaque monkeys by examining histochemical changes in the lateral geniculate nucleus and the striate cortex. They also discovered that the younger the age and the longer the animals lived, the faster and more severe the changes occurred. Long-term deprivation during the sensitive period can significantly alter the anatomical structure of the visual system, especially the level of synaptic connection in the visual cortex and lateral geniculate body. In a previous study, Mirzajani et al. (2011) observed similar alterations where induced myopia decreased the effect on occipital visual cortex activities. Moreover, a study conducted by Suzuki et al. (2015) indicated that refractive errors negatively affected visually evoked magnetic fields in V1. Therefore, we can conclude that monocular form deprivation can damage the ultrastructure of neurons in the visual cortex of rats during the sensitive period of visual development, which can be easily discovered using the VBM method.

In line with previous reports, we also observed reduced GMVs at the bilateral molecular layer of the cerebellum. The cerebellum is an crucial brain region involved in motor coordination. Although it constitutes only a small part of the brain, it contains 85% of all neurons in the human brain. The cerebellum has a complex neural structure that receives various motor and sensory inputs from the brain. It converts this diverse input information into output signals by using the same calculation principle and transmits them to the external structure of the cerebellum through the deep cerebellar nuclei. Research over the past two decades has found that the cerebellum also plays an essential role in non-motor functions. Schmahmann et al. (2019) summarized a large number of animal model tests and human functional magnetic resonance imaging results to find that the normal cerebellum is involved in cognition and emotion regulation. Peterburs et al. (2016) used functional magnetic resonance imaging to find that the cerebellum is related to eye movements. Another study observed two eye movement related regions in the cerebellum: the vestibular cerebellum and the vermis of the cerebellum (Thier and Markanday, 2019). Therefore, we conclude that the decrease in GMVs caused by form deprivation in the rat cerebellum may be associated with changes in cognition or eye movement.

By contrast, increased GMVs were found in the olfactory bulb of the FDM group. In the past, the olfactory bulb was thought to be the termination nucleus of the olfactory nerve and the primary center of the sense of smell. However, an increasing number of studies suggest that the olfactory bulb may participate in some visual activities. For instance, Schulze et al. (2017) demonstrated that prior to any olfactory stimulus, the piriform cortex preprocesses emotional visual information, and the emotional connotation of this preprocessing is then transmitted and incorporated into an extended olfactory network for olfactory processing. Fernandez et al. (Rosillo et al., 2013) discovered that connections between the olfactory and visual systems *via* the olfacto-retinal terminal nerve branch in Austrolebias charrua fishes suggest an early interaction between these sensory modalities. Lagnado et al. (Esposti et al., 2013) found that olfactory stimuli alter retinal sensitivity *via* dopaminergic regulation of presynaptic calcium channels, which control the gain of synaptic transmission *via* OFF bipolar cells. Philippa Johnson et al. (Andrews et al., 2022) studied the MRI results of 23 dogs and found that these MRI images presented nervous system connection between the occipital lobe and the olfactory bulb of dogs. Because of this connection, the dog’s brain can easily communicate between the two. There are also some insights into the functional enhancement of the olfactory bulb. For instance, electrophysiological recordings in the olfactory bulb in form deprived rats revealed stronger local field potentials than in normal ones (Zhou et al., 2017; Caravaca-Rodriguez et al., 2022). Therefore, the increased GMV of the olfactory bulb may compensate for the decreased visual cortex, which reflects compensatory or neural plasticity during the sensitive period of visual development in rats. In addition, cross-modal sensory communication, which is an intrinsic biological mechanism that relates to the interpretation of many forms of sensory input in the brain, occurred in conjunction with neuronal plasticity in rats.

Furthermore, an interesting finding in our results is that while the GMVs of the right subiculum, right cornu ammonis, and right entorhinal cortex were decreased, the right dentate gyrus and parasubiculum were increased. All of these brain regions belong to the hippocampus which is located between the cerebral thalamus and the medial temporal lobe. It consists of four regions: CA1, CA2, CA3 and CA4 (Yassa and Stark, 2011; Knierim, 2015; Zeidman and Maguire, 2016). The hippocampus is a part of the limbic system and is mainly responsible for the storage, conversion and orientation of short-term memory. Different regions of the hippocampus play a unique role in information processing, but to date, the specific functions of each region still need to be further studied. Wu et al. (Chan et al., 2017) used optogenetic activation technology, combined with pharmacological inhibition technology and functional MRI to stimulate the hippocampus with low-frequency activities, which can improve the functional activities of the cerebral cortex and can enhance visual responses by up to 20%. At the same time, other studies (Yau et al., 2012; Matsumoto et al., 2013) have indicated that visual stimulation can promote the regeneration of hippocampal neurons. In the case of the hippocampus of FDM rats, this change may be due to the change in visual stimuli, leading to the remodeling of hippocampal neurons to adapt to the current environment.

In this study, we observed positive NeuN and c-fos signals in the visual cortex of rats in both groups, indicating the presence of NeuN and c-fos expression. However, the IOD of NeuN and c-fos expression in the visual cortex of the FDM group was lower than that of the NC group. NeuN is an RNA-binding protein that is highly specific to mature neurons and is primarily expressed in their nuclei. Encoded by the FOX-3 gene, NeuN is recognized by monoclonal antibodies and widely used for neuron labeling and counting (Kuwabara et al., 2023). C-fos, a member of the early gene family, is closely associated with the onset and progression of myopia (He et al., 2018). Its expression in the visual cortex varies with changes in visual input during the critical period of visual development. Research has demonstrated that the c-fos gene is closely linked to myopia (Chen et al., 2017). Form-deprivation results in limited light stimulation of the visual cortex, leading to abnormal neuronal development and a subsequent reduction in intrinsic activity. This phenomenon may explain the decrease in GMV observed in the visual cortex of the FDM group compared to the NC group in the VBM analysis. Nevertheless, our study revealed a positive correlation between the mGMV and the expression of c-fos and NeuN of the visual cortex structure, suggesting that the molecular relationship between cortical activity and the macroscopic measurement of visual cortex structural plasticity exists. The decrease of NeuN, c-fos, and mGMV in the FDM group indicates that early form deprivation results in reduced numbers and activation of neurons in the visual cortex. To prevent the development of amblyopia caused by irreversible damage to neurons, it is necessary to remove form deprivation factors as soon as possible.

Nevertheless, some limitations of the present research need to be considered. For instance, a relatively small number of participants were enrolled, which may have affected reliability. Furthermore, this was simply a preliminary exploratory investigation comparing GMVs between the FDM group and the NC group. The next step should be to use a larger sample size to fully utilize the benefits of animal studies in conducting longitudinal research on the entire development process of myopia and perform a comprehensive study on the brain structure, functional connectivity, and diffusion tensor imaging of myopic rats using multimodal imaging. This will help gain a better understanding of the changes in brain structure and function that occur during various developmental stages, clarify the neural mechanism by which myopia develops, and identify potential methods for treating myopia.

According to this study, after monocular form deprivation, several brain regions of GMVs in the FDM group become atrophic or strengthened based on VBM analysis. We further speculate that form deprivation can lead to significant changes in specific brain regions. Changes in GMconcentration in the brain are typically interpreted as an indicator of cell size changes, neural or glial cell genesis, or apoptosis (Arentsen et al., 2017). Therefore, this change may function as a potential neuropathological mechanism in FDM. Further research is warranted to confirm this inference.

## 5. Conclusion

In summary, our study showed that FDM rats had altered brain structures in different regions, including the primary visual cortex and some extrastriate cortices, which are associated with brain remodeling due to visual function. This indicates that the visual cortex underwent developmental changes during the sensitive period of FDM rats’ visual development. We found a positive correlation between mGMV and the expression of c-fos and NeuN in the visual cortex, suggesting a molecular relationship between cortical activity and the macroscopic measurement of visual cortex structural plasticity. This finding may provide a useful supplement to understanding the anatomy of neural mechanisms in FDM. Additionally, our study confirms that the VBM method can be reliably used to analyze MRI data sets of rats and provides valuable supplementary information to clinical evaluations.

## Data availability statement

The raw data supporting the conclusions of this article will be made available by the authors, without undue reservation.

## Ethics statement

The animal study was reviewed and approved by Laboratory Animal Ethics Committee of Jinan University (Approval number: 20220512-16).

## Author contributions

JZ and JL: conception and design of the study and writing—original draft preparation. YHL and YML: analysis and interpretation. JL and YHL: MRI data process. QC: MRI data acquisitions. JL, YHL, and XT: rat modeling. JZ, YD, LL, and JL: writing—review and editing. JL: literature search. JL and YD: immunohistochemistry. All authors have read and agreed to the published version of the manuscript.

## Funding

This study was supported by the National Natural Science Foundation of China (Grant numbers: 81970806 and 82271094), Science and Technology Projects in Guangzhou (Grant number: 202201020030), and Medical Science and Technology Research Foundation of Guangdong (Grant number: A2021385).

## Conflict of interest

The authors declare that the research was conducted in the absence of any commercial or financial relationships that could be construed as a potential conflict of interest.

## Publisher’s note

All claims expressed in this article are solely those of the authors and do not necessarily represent those of their affiliated organizations, or those of the publisher, the editors and the reviewers. Any product that may be evaluated in this article, or claim that may be made by its manufacturer, is not guaranteed or endorsed by the publisher.

## Footnotes

^1^ www.fil.ion.ucl.ac.uk/spm
